# Prediction of drug resistance by Sanger sequencing of *Mycobacterium tuberculosis* complex strains isolated from multidrug resistant tuberculosis suspect patients in Ethiopia

**DOI:** 10.1371/journal.pone.0271508

**Published:** 2022-08-05

**Authors:** Eyob Abera Mesfin, Matthias Merker, Dereje Beyene, Abreham Tesfaye, Yassir Adam Shuaib, Desalegn Addise, Belay Tessema, Stefan Niemann

**Affiliations:** 1 Ethiopian Public Health Institute, National Laboratory Capacity Building Directorate, Addis Ababa, Ethiopia; 2 Molecular and Experimental Mycobacteriology, Research Center Borstel, Sülfeld, Germany; 3 Evolution of the Resistome, Research Center Borstel, Sülfeld, Germany; 4 Department of Microbial, Cellular and Molecular Biology, Addis Ababa University, Addis Ababa, Ethiopia; 5 Addis Ababa City Administration Health Bureau Health Research and Laboratory Services, Addis Ababa, Ethiopia; 6 College of Veterinary Medicine, Sudan University of Science and Technology, Khartoum North, Sudan; 7 Department of Medical Microbiology, College of Medicine and Health Sciences, University of Gondar, Gondar, Ethiopia; 8 German Center for Infection Research, Partner Site Hamburg-Lübeck- Borstel-Riems, Hamburg, Germany; The University of Georgia, UNITED STATES

## Abstract

**Background:**

Ethiopia is one of the high multidrug-resistant tuberculosis (MDR-TB) burden countries. However, phenotypic drug susceptibility testing can take several weeks due to the slow growth of *Mycobacterium tuberculosis* complex (MTBC) strains. In this study, we assessed the performance of a Sanger sequencing approach to predict resistance against five anti-tuberculosis drugs and the pattern of resistance mediating mutations.

**Methods:**

We enrolled 226 MTBC culture-positive MDR-TB suspects and collected sputum specimens and socio-demographic and TB related data from each suspect between June 2015 and December 2016 in Addis Ababa, Ethiopia. Phenotypic drug susceptibility testing (pDST) for rifampicin, isoniazid, pyrazinamide, ethambutol, and streptomycin using BACTEC MGIT 960 was compared with the results of a Sanger sequencing analysis of seven resistance determining regions in the genes *rpoB*, *katG*, *fabG-inhA*, *pncA*, *embB*, *rpsL*, *and rrs*.

**Result:**

DNA isolation for Sanger sequencing was successfully extracted from 92.5% (209/226) of the MTBC positive cultures, and the remaining 7.5% (17/226) *strains* were excluded from the final analysis. Based on pDST results, drug resistance proportions were as follows: isoniazid: 109/209 (52.2%), streptomycin: 93/209 (44.5%), rifampicin: 88/209 (42.1%), ethambutol: 74/209 (35.4%), and pyrazinamide: 69/209 (33.0%). Resistance against isoniazid was mainly mediated by the mutation *katG* S315T (97/209, 46.4%) and resistance against rifampicin by *rpoB* S531L (58/209, 27.8%). The dominating resistance-conferring mutations for ethambutol, streptomycin, and pyrazinamide affected codon 306 in *embB* (48/209, 21.1%), codon 88 in *rpsL* (43/209, 20.6%), and codon 65 in *pncA* (19/209, 9.1%), respectively. We observed a high agreement between phenotypic and genotypic DST, such as 89.9% (at 95% confidence interval [CI], 84.2%–95.8%) for isoniazid, 95.5% (95% CI, 91.2%–99.8%) for rifampicin, 98.6% (95% CI, 95.9–100%) for ethambutol, 91.3% (95% CI, 84.6–98.1%) for pyrazinamide and 57.0% (95% CI, 46.9%–67.1%) for streptomycin.

**Conclusion:**

We detected canonical mutations implicated in resistance to rifampicin, isoniazid, pyrazinamide, ethambutol, and streptomycin. High agreement with phenotypic DST results for all drugs renders Sanger sequencing promising to be performed as a complementary measure to routine phenotypic DST in Ethiopia. Sanger sequencing directly from sputum may accelerate accurate clinical decision-making in the future.

## Background

Tuberculosis (TB) is still a major public health problem with 10 million incident cases and 1.5 million TB deaths in 2019 globally, of which 24% of the cases are reported from Africa [1]. Efforts to control TB have been confronted by the emergence and transmission of drug-resistant MTBC strains in many geographical areas (e.g., developing countries) [2]. Multidrug-resistant tuberculosis (MDR-TB) is one of the major global threats and is defined as resistance to at least rifampicin (RIF) and isoniazid (INH). According to the WHO report, 3.4% of new and 18% of previously treated cases had MDR-TB or RIF resistant (RR)-TB worldwide, and 2.6% of new and 11% of previously treated cases were estimated to have MDR-TB/RR-TB in Africa [1].

Ethiopia is one of the countries with the highest TB, TB/HIV, and MDR TB burdens, with an estimated national TB incidence of 132 per 100,000 population and 108,714 notified new and relapse cases in 2019 [1, 2]. According to WHO, the prevalence of MDR/RR TB was estimated at 0.71% in new cases and 12% in previously treated cases [1]. Despite this, studies conducted in the country revealed that the prevalence of MDR-TB ranged from 5% in the Northwestern part of the country to 46.3% in the central part (i.e., Jima and Addis Ababa) [3–6]. Moreover, our published report from this cohort population showed that the prevalence of MDR-TB among MDR-TB suspect patients in Addis Ababa, Ethiopia was 39.4%, with more than 58% of these patients being resistant to all first-line TB drugs [7].

Drug resistance in MTBC strains arises from mutations in functional genes [8]. These mutations often lead to changes of specific protein regions, e.g. drug binding sites, or occur in promoter regions of genes, resulting in increased transcription [8]. For instance, RIF resistance is associated with mutations found in an 81 bp "hot-spot" region of the gene *rpoB*, including codons 507 to 533 [9, 10]. Mutations associated with INH resistance occur mainly in the gene *katG* that encodes for a catalase-peroxidase enzyme activating the drug or in the promoter region of the *fabG1/inh*A operon, which increases the transcription of the drug target protein *(InhA)* [10, 11]. While mutations in the genes *rpsL*, *rrs*, and *gidB* can confer resistance to streptomycin (STR), resistance to ethambutol (EMB) is mediated by mutations found in *embB* [11–13]. Moreover, mutations in the gene *pncA* are associated with resistance to pyrazinamide (PZA) [11–13].

Accurate and rapid drug susceptibility testing (DST) is crucial for appropriate TB treatment [14]. However, the use of phenotypic DST (pDST) is confined to reference or central laboratories in many developing countries [15]. Molecular assays or genotypic DST (gDST), such as Cepheid GeneXpert and Hain MTBDRplusv2.0, on the other hand, interrogate only a few canonical mutations. Thus, it is important to identify which mutations are most prevalent in Ethiopia. For instance, resistance mediating mutations that are not interrogated by commercial molecular tests may lead to false negative results, or particular combinations of mutations may lead to false resistant interpretations [15].

Therefore, the aim of this study was to characterize mutations associated with resistance against first-line anti-TB drugs in MTBC strains isolated from suspected MDR-TB patients in Addis Ababa, Ethiopia, and to compare the performance of DNA-sequencing for detection of resistance in comparison to the routine phenotypic DST method.

## Materials and methods

### Study design and setting

A cross-sectional study was conducted from June 2015 to December 2016 in all health facilities that provide MDR-TB diagnosis services in Addis Ababa city, namely Addis Ababa Regional Referral Laboratory, Saint Peter Hospital, and Teklehaimnot Health Center. We enrolled 226 MDR-TB suspect cases who were culture positive and consented to participate in the study, including TB treatment failure cases, smear-positive cases who had known close contact with a confirmed MDR-TB patient, and new or retreatment cases who remained smear-positive for at least two or three months of treatment, respectively [16].

**Fig 1:**
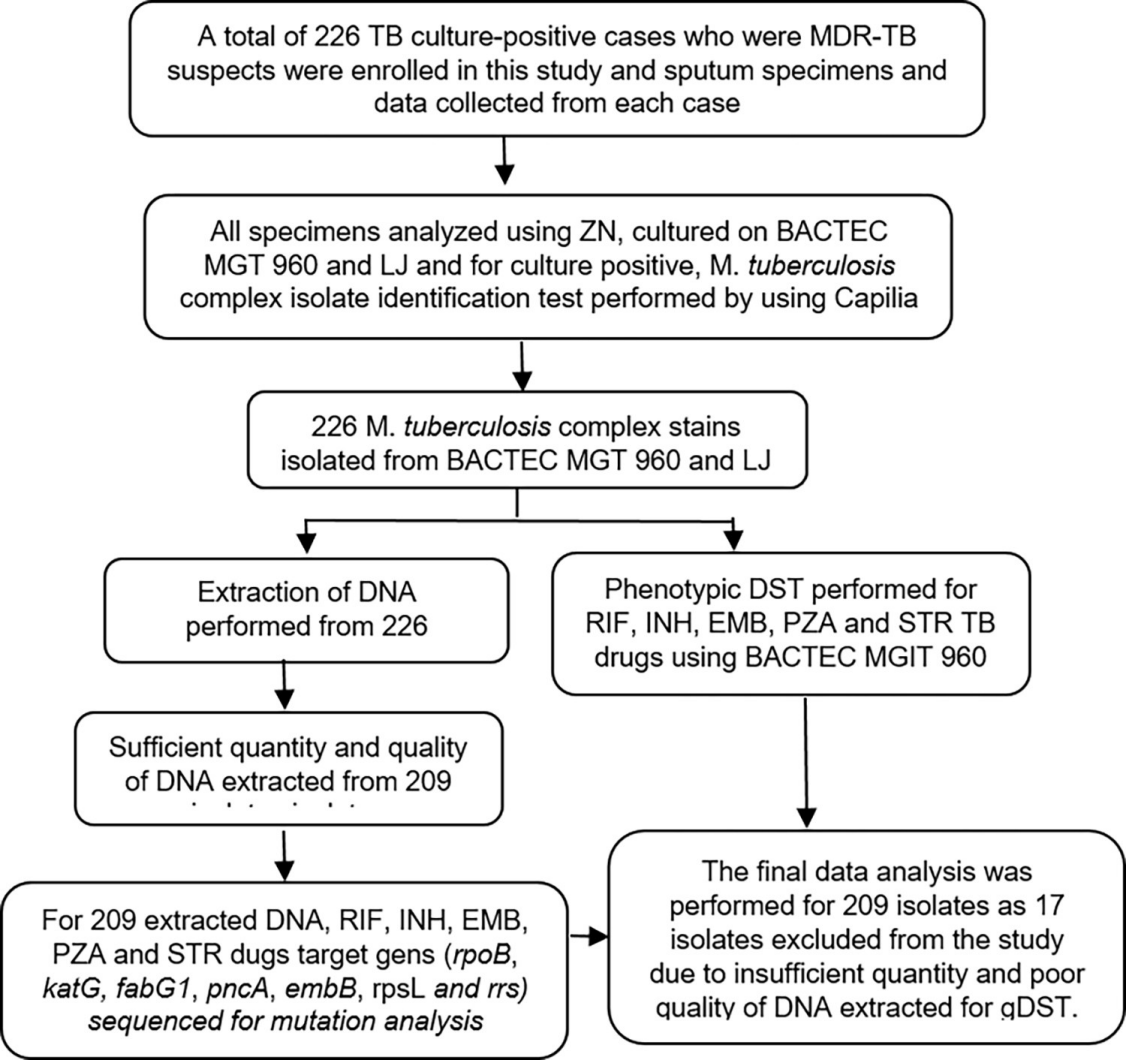
A flowchart explaining the steps of the study.

Besides sputum specimens, we collected socio-demographic, epidemiological, and clinical data from each study participant using a questionnaire. Mycobacterial culture and pDST were performed at the Ethiopian Public Health Institute, National Reference TB Laboratory, whereas Sanger sequencing was performed at the Research Center Borstel in Germany (Fig 1).

### Specimen collection and laboratory analysis

#### Specimen collection

A minimum volume of 5 ml of sputum specimen produced by a deep cough was collected into a sterile wide mouth 50 ml falcon tube from each study participant. All specimens were stored at 2–8°C at collection sites until transported to the National TB Reference Laboratory using a cold chain [7].

#### Microscopy examination

All collected samples were subjected to Ziehl-Neelsen (ZN) staining as described previously [7]. Briefly, a smear was prepared using a slide from the mucopurulent part of the sputum, air-dried, and stained. The stained slides were examined using a light microscope for the presence of Acid Fast Bacilli (AFB) [17].

#### Specimen decontamination and culture

For better yield, Lowenstein Jensen (LJ) and Mycobacteria Growth Indicator Tube (MGIT) culture methods were used. All sputum samples were decontaminated with 4% sodium hydroxide-N-acetyl-l-cysteine (NaOH-NALC) and then neutralized with phosphate-buffered saline (PBS). The decontaminated samples were then inoculated into Mycobacteria Growth Indicator Tubes (MGIT BACTEC™ MGIT 960 tubes (BD Diagnostics, Sparks, MD, USA) at 37°C [18], and onto LJ slants at 37°C [19]. The incubated specimens in the BACTEC™ MGIT 960 tube were inspected daily for 42 days maximum to check growth [18]. Similarly, an inspection of the specimens incubated in LJ media was done weekly for eight weeks based on colony growth and morphology [19].

#### Identification of mycobacteria

Identification of the grown mycobacteria species was done by using MPT64 antigen detection methods (Capilia TB-neo Becton, Dickinson Diagnostic Systems; Sparks, MD, USA). Briefly, the test device consisted of a sample area, a test area containing the anti-MPB64 antibodies, and a control area where anti-species immunoglobulin antibodies are fixed. The testing method is based on immune-chromatographic principles, in which antibodies labeled with colloidal particles react with target antigens to form a migrating antigen-antibody complex, which is captured by a second fixed antibody. A color reaction takes place when the labeled particles are fixed. The result is interpreted as positive for the MTBC if the color reaction takes place in the test and control areas [20].

#### Phenotypic drug susceptibility testing

The DST for RIF, INH, EMB, STR, and PZA was performed using the BACTEC™ MGIT 960 method as described previously [7]. Briefly, 0.1 ml of a bacilli suspension with a McFarland standard was inoculated into a vial supplemented with reconstitution solution, and containing 1.0 μg/ml of RIF, 0.1 μg/ml of INH, 5.0 μg/ml of EMB, 1.0 μg/ml of STR, and 100 μg/ml of PZA [18]. *Mycobacterium tuberculosis* strain H37Rv was used as a sensitive control for susceptibility testing. The result was interpreted when the growth unit value of the growth control reached 400 or more within 4 to 13 days. If the growth unit value of the tube containing the drug being tested was 100 or more, the strain was classified as resistant; if the growth unit value was less than 100, the strain was classified as susceptible.

#### Genomic DNA extraction

Genomic DNA was extracted from MTBC strains by a method described by Somerville et al. [21]. Briefly, a loop full of MTBC colonies was suspended in 400 μl of 10 mM Tris–HCl, 1 mM ethylene-diamine-tetra-acetic acid (EDTA) and heated for 20 minutes at 80°C. Then 1 mg/ml of lysozyme was added and incubated for 2 hours at 37°C. This was followed by the addition of proteinase K (0.2 mg/ml) and 10% sodium dodecyl sulfate in distilled, deionized water (1.1%) and incubated at 65°C for 20 minutes after vortex. After incubation, a mixture of N-acetyl-N, N, N-trimethyl ammonium bromide [40 mM], and NaCl (0.1 M) was added, and then NaCl (0.6 M) was immediately added. The mixture was vortexed until it turned milky and incubated at 65°C for 10 minutes. A 750 μl chloroform-isoamyl alcohol (24:1) was added, vortexed, and then centrifuged at 13,000 rpm in a microcentrifuge for 5 minutes at room temperature. Then the extracted DNA was precipitated with 70% ethanol and re-suspended in a volume of 30 μl TE buffer. Finally, DNA quality and concentration were determined by a spectrophotometer at an optical density of 260 nm and 280 nm.

### Polymerase Chain Reaction and drug target gene sequencing

Polymerase Chain Reaction (PCR) amplification and sequencing of the RIF, INH, EMB, PZA, and STR drugs’ targets in MTBC strains was done by using gene-specific primers as described below in Table 1. The PCR reactions were conducted in a volume of 25 μL final reaction mix of 2.5 μl of 10x PCR buffer (10 mM Tris-HCl, pH 8.3; 50 mM KCl; 0.001% gelatin), 0.5 μl of 0.2 mM dNTPs, 0.75 μl of 25 mM magnesium chloride solution, 0.125 μl of 2 U AmpliTag Gold polymerase (Perkin Elmer, USA), 1.25 μl each of the 10 μM primers (forward and reverse primers), 1.25 μl of DMSO, 15.3 μl of double distilled water and 2 μl of genomic DNA. The amplification was done by programming the thermocycler of Eppendorf™ at the following conditions: 95°C for 3 minutes for initial denaturation; followed by 40 cycles of denaturation at 95°C for 1 minute, annealing ranged from 55°C to 65°C for 30 seconds or 1 minute (summarized in Table 1 for each gene), and extension at 72°C for 30 seconds, and the final extension was at 72°C for 5 minutes. The PCR amplified products were examined on a 1.5% agarose gel electrophoresis using a 100 base pair DNA ladder.

**Table 1:** Primers that were used for PCR amplification and sequence of drug target genes for analysis of the mutation in MTBC strains.

Gene	Primer Sequence (5’→3’)		Amplicon size	Annealing (Time)	Reference
*rpoB*	Forward	TCGCCGCGATCAAGGAGT	157bp	65°C (30 sec)	[22]
	Reverse	GTGCACGTCGCGGACCTCCA			
*katG*	Forward	TCGGCGATGAGCGTTACAGC	543bp	65°C (30 sec)	[23]
	Reverse	CCCGCAGCGAGAGGTCAGTGG			
fabG1-inhA	Forward	CCTCGCTGCCCAGAAAGGGA	230bp	55°C (1 min)	[24]
	Reverse	ATCCCCCGGTTTCCTCCGGT			
*rpsL*	Forward	CGGCGGGTATTGTGGTTGCTCGTG	801bp	55°C (1 min)	[25]
	Reverse	CCTCCAGGGCGGGTTTGACATTG			
*rrs*	Forward	CCATTGCCGGATTTGTATTAGACT	843bp	55°C (1 min)	[26]
	Reverse	GCGGGCGATACGGGCAGACTA			
*embB*	Forward	TGGACGGGCGGGGCTCAAT	334bp	65°C (30 sec)	[22]
	Reverse	CCAGCGCCGCCGGTGTGAGC			
*pncA*	Forward	GCTGGTCATGTTCGCGATCG	665bp	60°C (30 sec)	[27]
	Reverse	CGCTCCACCGCCGCCAACAG			

Finally, EXOSAP cleanup of PCR products for sequencing was performed under the following conditions: 5 μl PCR products were mixed with 1μl exonuclease and 1μl alkaline phosphatase, and then the mix was placed in a thermal cycler with the hot lid off. The cycles were performed for 30 min at 37°C and 15 min at 80°C [22], and followed by a Sephadex cleanup of the sequence-PCR products. The resulting products were sequenced with their gene-specific forward and reverse single primer extensions to get optimal coverage of the target regions using a Big dye-terminator kit and an ABI Prism 3500lL Genetic Analyzer (Applied Biosystems, USA).

### Data analysis

The sequencing data obtained from the ABI3730XL DNA analyzer were imported into SeqScape® software version 2.7 (Applied Biosystems, Foster City, CA) and consensus sequences were generated. The SeqScape® was used for DNA sequence comparisons, and mutations were detected in the respective genes by comparing them with the reference *Mycobacterium tuberculosis* strain H37Rv sequence. Likewise, all patient-related information collected, phenotypic drug profiles, and drug target gene mutation data were compiled, entered into an excel sheet, cleared, and categorized as necessary. Descriptive statistics were computed, including frequency and percentage of the socio-demographic, TB exposure and treatment history, antibiotic treatment history, HIV status, alcohol consumption and smoking history, phenotypic drug profiles, and mutations identified from drug target gene data using SPSS version 23 statistical package software (SPSS Inc., Chicago, IL).

### Performance of Sanger sequencing for the prediction of drug resistance

Sensitivity, specificity, and overall agreement were calculated in comparison to the phenotypic DST results from the reference standard BACTEC MGIT960 (Becton Dickinson). Any identified mutation in the selected resistance determining regions (Table 1) was considered a genotypic drug resistance determinant. Sensitivity was calculated as the number of true positives divided by the number of true positives plus the number of false positives. Calculating specificity was done by dividing the number of true negatives by the number of true negatives plus the number of false positives. Overall agreement was calculated as the number of true positives plus the number of true negatives divided by the number of all examined samples (true negatives and positives plus false negatives and positives).

### Ethical considerations

Scientific and ethical approval for the study was obtained from the Research and Ethical Review Committee of Addis Ababa University and the Ethiopian Public Health Institute. We obtained written and/or oral informed consent from study participants. Confidentiality of the participants’ data and test results was maintained throughout the study period using codes.

## Results

Overall, we enrolled a total of 226 MTBC MDR-TB suspected cases and successfully isolated MTBC strains from all cultured samples (100%). However, from these, we were able to extract DNA with enough quantity and quality for gDST from 209 (92.5%) strains. Therefore, we excluded 17 strains (study participants) from the final analysis of this study.

### Socio-demographic and clinical characteristics

Some socio-demographic and clinical characteristics data of the study participants used in this report were included in our previous report [7]. As shown in Table 2, the majority of MDR-TB suspects were males (59.3%, 124/209), married (59.3%, 124/209), and HIV positive (58.9%, 123/209). The largest age group of patients was between 24 and 34 years old (94/209, 45.0%). Of note, 86.6% (181/209) of the study participants were ZN smear positive. The majority of the investigated patients had a previous TB treatment history (70.8%, 148/209). Out of these, 90.5% (134/148) were relapsed cases and 9.5% (14/209) were treatment failures and defaults. Seventy-nine (79/209, 37.8%) cases had an antibiotic treatment history, and more than one-third (28/79, 35.4%) of these cases interrupted antibiotic treatment more than once. Moreover, 41/209 (19.6%) of the participants reported frequent alcohol consumption and 26/209 (12.4%) cigarette consumption (Table 2).

**Table 2:** Socio-demographic and TB-related characteristics of MDR-TB suspected and confirmed cases.

Variable	All DR-TB suspected Cases Number (%) (n = 209)	MDR-TB confirmed Cases Number (%) (n = 88)
**Sex**		
Male	124 (59.3)	37 (42.0)
Female	85 (40.7)	51 (58.0)
**Age Group**		
15–24	25 (12.0)	4 (4.5)
25–34	94 (45.0)	53 (60.2)
35–44	62 (29.7)	22 (25.0)
45–54	19 (9.1)	6 (6.8)
Above 54	9 (4.3)	3 (3.4)
**Marital Status**		
Married	124 (59.3)	53 (60.2)
Unmarried	75 (35.9)	31 (35.2)
Divorced	7 (3.3)	2 (2.3)
Widow	3 (1.4)	2 (2.3)
**Living Region**		
AA	193 (92.3)	82 (93.2)
Amhara	1 (0.5)	0 (0)
Dire Dawa	2 (1.0)	2 (2.3)
Oromia	11 (5.3)	4 (4.5)
SNNPR	2 (1.0)	0 (0)
**Residence**		
Rural	12 (5.7)	4 (4.5)
Urban	197 (94.3)	84 (95.5)
Previously TB infected		
No	61 (29.2)	16 (18.2)
Yes	148 (70.8)	72 (81.8)
Treatment history of previously TB infected cases		
No	0 (0)	0 (0)
Yes	148 (100)	72 (100)
Treatment interruption previously TB treated cases		
No	126 (85.1)	62 (86.1)
Yes	22 (14.9)	10 (13.9)
TB Treatment History		
New	61 (29.2)	16 (18.2)
Previously treated	148 (70.8)	72 (81.8)
Previously treated cases ((among retreatment cases)		
Defaulter	5 (3.4)	3 (4.2)
Relapse	134 (90.5)	62 (86.1)
Treatment Failure	9 (6.1)	7 (9.7)
ZN Microscopy Results		
Negative	28 (13.4)	2 (2.3)
Positive	181 (86.6)	86 (97.7)
HIV Status		
Positive	123 (58.9)	70 (79.5)
Negative	86 (41.1)	18 (20.5)
Antibiotic treatment history		
No	130 (62.2)	45 (51.1)
Yes	79 (37.8)	43 (48.9)
Antibiotic treatment interruption (among treated cases)		
No	51 (64.6)	28 (65.1)
Yes	28 (35.4)	15 (34.9)
Alcohol drinking frequently		
No	168 (80.4)	60 (68.2)
Yes	41 (19.6)	28 (31.8)
Alcohol drinking during treatment (among drinkers)		
No	32 (78.0)	22 (78.6)
Yes	9 (22.0)	6 (21.4)
Cigarettes Smoking		
No	183 (87.6)	72 (821.8
Yes	26 (12.4)	16 (18.2)

SNNPR: Southern Nations, Nationalities, and Peoples’ Region, HIV: Human Immunodeficiency Virus ZN: Ziehl-Neelsen

### Phenotypic drug susceptibility tests

The pDST data used herein was included in our previously published report [7]. Table 3 shows the details of all the pDST results and the identified resistance mediating mutations. Overall, 122/209 (58.4%) of the strains showed resistance to at least one of five anti-TB drugs tested (RIF, INH, PZA, EMB, and STR), and the proportion of resistance to INH, STR, RIF, EMB, and PZA was 109 (52.2%), 93 (44.5%), 88 (42.1%), 74 (35.4%), and 69 (33.0%), respectively. Of these, 88 (72.1%) stains were MDR-TB and the remaining 34/122 (27.9%) strains had mono or polydrug resistance. Interestingly, 53/88 (60.2%) MDR-TB strains were resistant to all five anti-TB drugs (RIF, INH, PZA, EMB, and STR), as shown in Table 3. Most of the MDR TB strains were from ZN smear-positive cases, i.e., 86/88 (97.7%) [Table 2].

**Table 3:** Phenotypic and genotypic drug resistance patterns in MTBC strains.

Phenotypic Pattern of Drug Resistance		Genotypic Pattern of Drug Resistance	
Drug Resistance	All strains (n = 209) n (%)	Drug target Genes Mutation	All strains (n = 209) n (%)
**Any Resistance**	**122 (58.4)**	**Any Mutation**	**110 (52.6)**
INH	109 (52.2),	*KatG*	100 (47.8)
RIF	88 (42.1)	*rpoB*	90 (43.1)
STR	93 (44.5)	*fabG-inhA*	7 (3.3)
EMB	74 (35.4),	*embB*	73 (34.9)
PZA	69 (33.0)	*pncA*	68 (32.5)
		*rpsL*	49 (23.4)
		*rrs*	10 (4.8)
**Mono Resistance**	**16 (7.7)**	**Single Mutation**	**13 (6.2)**
INH	8 (3.8)	*rpoB*	5 (2.4)
STR	7 (3.3)	*KatG*	5 (2.4)
PZA	1 (0.5)	*rpsL*	2 (1.0)
		*fabG-inhA*	1 (0.5)
**Multi drug Resistance (MDR)**	**88 (42.1)**	**Multiple Mutation with MDR**	**85 (40.7)**
RIF + INH	3 (1.4)	*rpoB* + *KatG*	5 (2.4)
RIF + INH + EMB	3 (1.4)	*rpoB* + *KatG* + *embB*	5 (2.4)
RIF + INH + STR	7 (3.3)	*rpoB* + *KatG* + *rpsL*	5 (2.4)
RIF + INH + PZA	4 (1.9)	*rpoB* + *KatG* + *pncA*	6 (2.9)
RIF + INH + EMB + STR	10 (4.8)	*rpoB* + *KatG* + *pncA* + *rpsL*	4 (1.9)
RIF + INH + EMB + PZA	3 (1.4)	*rpoB* + *KatG* + *embB* + *rpsL*	5 (2.4)
RIF + INH + STR + PZA	5 (2.4)	*rpoB* + *KatG* + *embB* + *pncA*	19 (9.1)
RIF + INH + EMB + STR + PZA	53 (25.4)	*rpoB* + *KatG* + *fabG-inhA* + *embB* + *pncA*	2(1.0)
		*rpoB* + *KatG* + *embB* + *pncA* + *rrs*	3(1.4)
		*rpoB* + *KatG*+ *embB* + *pncA* + *rpsL*	26 (12.5)
		*rpoB* + *KatG* + *embB* + *pncA* + *rpsL* + *rrs*	2 (1.0)
		*rpoB* + *KatG* + *fabG-inhA* + *embB* + *pncA* + *rpsL*	2 (1.0)
		*rpoB* + *KatG* + *fabG-inhA* + *embB* + *pncA* +*rrs*	1 (0.5)
Poly Resistance* (Non MDR)	**14 (6.7)**	**Multiple Mutation without MDR**	**9 (4.3)**
EMB + INH	3 (1.4)	*rpoB* + *embB* + *pncA* + *rrs*	1 (0.5)
INH + STR	6 (2.9)	*KatG*+ *embB* + *pncA*	1 (0.5)
EMB + INH + STR	2 (1.0)	*KatG*+ *embB* + *rpsL*	1 (0.5)
INH + STR + PZA	2 (1.0)	*KatG*+ *embB*	3 (1.4)
EMB + INH + STR + PZA	1 (0.5)	*KatG*+ *rpsL*	2 (1.0)
		*KatG*+ *pncA*	1 (0.5)

### Genotypic drug susceptibility testing

A total of 110/209 (52.6%) strains had a mutation either in the *rpoB*, *KatG*, *fabG-inhA*, *embB*, *pncA*, *rpsL*, and or *rrs* genes. Of these, 85/209 (40.7%) strains were confirmed as MDR-TB (Table 3). Mutations in the *rpoB* associated with resistance against RIF were detected in 90/209 (43.1%) of the strains, while the mutation *rpoB* S531L was most prevalent (57/90, 63.3%). In 15/90 (16.7%) of the strains with Rifampicin Resistant Determinant Region (RRDR) mutations, codon 526 was affected by different mutations (H526Y, H526S, H526D, and H526L). Other mutations, including the *rpoB* D516V, Q513P, L533P, and L538P/V, were also observed, in addition to one base pair insertion at codon 513.

Among all the genotypic RIF resistant strains, 84/90 (93.3%) were also phenotypically resistant at 1.0 μg/ml (MGIT960). However, the six phenotypically susceptible strains harbored mutations in the *rpoB* R529P (one strain), L533P (two strains), and L538PV (two strains), and one strain had a silent mutation, i.e., S531S (TCG to TCC). Moreover, four phenotypic resistant strains did not have any mutations in the interrogated *rpoB* gene region (Table 4).

**Table 4:** Mutations associated with drug resistance in the *rpoB* gene, *katG* gene, *fabG-inhA* promoter region, *pncA gene*, *embB gene*, *rpsL gene and rrs gene* of MTBC strains.

*rpoB* Gene (RIF drug target Gene)				Kat G Gene (INH drug target Gene)				fabG1-inhA*** (INH drug target Gene)				*pncA* Gene (PZA drug target Gene)				*embB* Gene (EMB drug target Gene)				*rpsL* Gene (STR drug target Gene)				*rrs* Gene STR drug target Gene)			
Codon & Amino acid Change	Nucleotide Change	No. of strains with mutation (n = 90), n (%)	No. of pDST susceptible strains with mutation	Codon & Amino acid Change	Nucleotide Change	No. of strains with mutation (n = 100), n (%)	No. of pDST susceptible strains with mutation	Codon & Amino acid Change	Nucleotide Change	No. of strains with mutation (n = 5), n (%)	No. of pDST susceptible strains with mutation	Codon & Amino acid Change	Nucleotide Change	No. of strains with mutation (n = 68), n (%)	No. of pDST susceptible strains with mutation	Codon & Amino acid Change	Nucleotide Change	No. of strains with mutation (n = 74), n (%)	No. of pDST susceptible strains with mutation	Codon & Amino acid Change	Nucleotide Change	No. of strains with mutation (n = 49), n (%)	No. of pDST susceptible strains with mutation	Codon & Amino acid Change	Nucleotide Change	No. of strains with mutation (n = 10), n (%)	No. of pDST susceptible strains with mutation
513 insA	A inserted	1 (1.1)		S315T	A**G**C to ACC	95 (95)	2 (2.0)	**-15**	C- to T	4 (57.1)	1 (14.3)	65fs	193 Ins A	19 (27.9)		M306I**	ATG→ ATA	17 (23.0)		K88R****	AAG→AGG	22(44.9)	2 (4.0)	514 ^¥^	A → C	2(20)	
Q513P	CAA to CCA	3 (3.3)		S315H	AGC to AG**A**	1 (1.0)	** **	**-8**	T- to C	1 (14.3)		V130G	GTG→ GGG	5 (7.4)	1 (1.5)	M306I, 347** fs	ATG→ ATA, 1039 del A	1 (1.4)		K88T*****	AAG→ACG	21 (42.9)	1 (2.0)	517	C → T	2(20)	
D516V	GAC to GTC	4 (4.4)		S315T, V14G**	A**G**C to ACC GTA to GGA,	1 (1.0)	** **	** **				T76P	ACT→ CCT	4 (5.9)		M306I, G406A**	ATG→ ATA, GGC→GCC	1 (1.4)		K43R	AAG→AGG	6 (12.2)		631***,^¥^	A →C*****	1(10)	
S522L	TCG to TG	1 (1.1)		S315T G28R**	A**G**C to ACC GGG to CGG	1 (1.0)	** **	** **				A102V	GCA→GTA	4 (5.9)	3 (4.4)	M306I, G406D**	ATG→ ATA, GGC→GAC	1 (1.4)						891 **^, ¥^	G →A****	2(20)	
H526Y	CAC to TAC	9 (10.0)		E334Q	GAG to CAG	1 (1.0)	** **	** **				V139A	GTG→GCG	4 (5.9)		M306I	ATG→ATC	5 (6.8)						906 ^¥^	A → G	2(20)	1(10)
H526Y, S531L**	CAC to TAC, TCG to TTG,	1 (1.1)		221	661–662 insertion G	1 (1.0)						Y41Stop	TAC→TAG	2 (2.9)		M306V	ATG→ GTA	21 (28.4)						1010^¥^	A →C	1(10)	
H526D	CAC to GAC	2 (2.2)		** **								Y103H	TAC→ CAC	2 (2.9)		M306L	ATG→ CTA	2 (2.7)									
H526L	CAC to CTC	1 (1.1)		** **								V130M	GTG→ATG	2 (2.9)	1 (1.5)	D311G	GAC→GGC	1 (1.4)	1 (1.4)								
H526S	CAC to AAC	2 (1.1)		** **								V44G	GTC → GGC	1 (1.5)		M316R, D354A**	ATG→AG, GAT→ GCT	1 (1.4)									
R529P	CGA to CGC	1 (1.1)	1 (1.1)									A46T	GCA→ACA	1 (1.5)		D328H, D35AD**	GAT→ CAT GAT→GCT	1 (1.4)									
S531L	TCG to TTG	56 (62.2)										K48E	AAG→GAG	1 (1.5)		W332G	TGG→GGG	1 (1.4)									
S531L, R548**	TCG to TTG, CGC to CGG	1 (1.1)										H57Y	CAC→TAC	1 (1.5)		S347C, 402** fs	AGT→TGT, 1204 del C	1 (1.4)									
S531W	TCG to TGG	3 (3.3)										I6T	ATC→ACC	1 (1.5)		E378A, G406D**	GAG→GC, GGC→GAC	1 (1.4)									
S531S*	TCG to TCC	1 (1.1)	1 (1.1)*									C72R	TGC →CGC	1 (1.5)		368 fs	1101–1102 ins G	1 (1.4)									
L533P	CTG to CCG	2 (2.2)	2 (2.2)									G78S	GGC→AGC	1 (1.5)		G406A	GGC→GCC	13 (17.6)									
L538P	CTG to CCG	1 (1.1)	1 (1.1)									100–101, fs	300–301 ins GC	1 (1.5)		A409P	GCG→CCG	1 (1.4)									
L538V	CTG to GTG	1 (1.1)	1 (1.1)									G108A	GGA→GCC	1 (1.5)		347 fs	1039 del A	4 (5.4)									
												G108E, 183, fs**	GGA→GAA, 547 del G	1 (1.5)													
												W119R	TGG→CGG	1 (1.5)													
												W119 Stop	TGG→TGA	1 (1.5)													
												W119C	TGG→TGT	1 (1.5)													
												D12A	GAC→ GCC	1 (1.5)													
												L182W	TTG→ TGG	2 (2.9)													
												-11	A→ G	2 (2.9)													
												V7G	GTC→GGC	1 (1.5)													
												127, fs	380–388 del AGGTCGATG	1 (1.5)													
												D129N	GAT→AAT	1 (1.5)													
												C138W	TGT→TGG	1 (1.5)													
												V155A	GTG→ GCG	1 (1.5)													
												T160P	ACA→CCA	1 (1.5)													
												177, fs	530 ins A	1 (1.5)													
												V180F	GTC → TTC	1 (1.5)													

**fs: frameshift**, **del: deletion, ins: insertion;**

* Silent mutation

** Gene that has multiple mutations

*** all MDR-TB strains that had mutation in *fabG1*-inhA have additional mutation in *katG* codon 315

**** Multiple mutations were found in *rpsL* (K88R) and *rrs* (891, G → A)

***** Multiple mutations were found in *rpsL* (K88T) and *rrs* (631, A → C), ¥ numbers indicated nucleotide position

A total of 100/209 (47.9%) strains had a mutation in the *katG* gene, with 98/100 (98.0%) strains harboring the *katG* S315T mutation and 1/100 (1%) strains having a double mutation, i.e., *katG* S315T and T275A. Moreover, mutations in the promoter region of the INH drug target *(InhA*) were identified in 5/209 (2.4%) strains, i.e., -8 t/c and -15 c/t relative to the gene start. Interestingly, most 4/5 (80.0%) of these strains also had a mutation *katG* S315T, mediating high level INH resistance (Table 4). Likewise, 2/209 (1.0%) strains had other distinct mutations in *katG* in combination with *katG* S315T, i.e., V14G & S315T, and G28R & S315T.

Genotypic EMB resistance was detected in 74/209 (35.4%) strains mediated by mutations in the *embB* gene. The most common mutations were observed at codon 306 in 48/74 (64.9%) of the strains, and of these, 66/74 (89.2%) of the *embB* mutations were found in MDR-TB strains (Table 4). Additional mutations associated with EMB resistance were detected in 13/74 (17.6%) strains with *embB* G406A (GGC to GCC), 1/74 (1.4%) strains with a frameshift mutation at codon 368, and 4/74 (5.4%) strains with a frameshift at codon 347. Moreover, other unique mutations in *embB* were observed in 7/74 (9.5%) strains (Table 4).

With regard to PZA resistance, a total of 68/209 (30.1%) strains had a mutation in the *pncA* gene. Among PZA susceptible strains, we identified 5/209 (2.4%) strains with *pncA* A102V, one strain with *pncA* V130G, and one strain with *pncA* V130M. Of note, 5/209 (2.4%) strains with *pncA* V130G and 2/209 (1.0%) strains with *pncA* V130M tested PZA resistant in MGIT960.

The most prevalent mutation was a single base pair insertion at position 193, which was found in 19/209 (9.1%) of the strains. Other mutations were identified in 20/209 (9.6%) strains as follows: *pncA* T76P, A102V and V139A, Y41Stop, Y103H, G108E, G108A, and L182W. Moreover, the following frameshift mutations were found in *pncA* 300insG, 301insC, 380–388del AGGTCGATG, and 530delA. All *pncA* mutations co-occurred in strains that had mutations in *rpoB*, *katG*, *fabG1*, *embB*, *rpsL*, or *rrs* (Table 3).

Mutations associated with resistance to STR were detected in 57/209 (27.3%) of the strains, and the most prevalent mutations were *rpsL* K88R 22/209 (10.5%) and the *rpsL* K88T 21/209 (10.0%), and the *rpsL* K43R 7/209 (2.9%). Furthermore, mutations in the *rrs* gene were identified in 10/209 (4.8%) strains at positions 514 (A to C), 517 (C to T), 613 (A to C), 891 (G to A), 906 (A to G), and 1010 (A to C). Overall, 43/57 (87.8%) strains with mutations in *rpsL* had an additional mutation in *rpoB* and *katG*, i.e., co-occurred with an MDR genotype.

### Performance of Sanger sequencing

We further investigated the sensitivity and specificity of the prediction of individual drug resistances and overall agreement (proportion of resistant and susceptible strains) of Sanger sequencing DST for RIF, INH, PZA, EMB, and STR, compared to the phenotypic standard method BACTEC™ MGIT 960 as described in Table 5. Our finding showed that the sensitivity and specificity for RIF were 95.5% with a 95% confidence interval (CI) of 91.2% to 99.8% and 95.9% (95%, CI, 92.4% to 99.4%), respectively, resulting in a concordance of 95.7% (95%, CI, 92.9% to 98.5%). Six discordant resistant results were linked to the mutations in *rpoB* R529P, L533P, L538P/V, and S531S (silent mutation) (Table 4).

**Table 5:** Sensitivity, specificity, and overall accuracy of gDST results to predict resistance against RIF, INH, PZA EMB, and STR.

		Phenotypic DST Result using BACTEC™ MGIT 960									
		RIF		INH		PZA		EMB		STR	
		R	S	R	S	R	S	R	S	R	S
Genotypic DST using Sanger Sequencing	R	84	6	98	2	63	5	73	1	53	4
	S	4	115	11	98	6	135	1	134	40	112
	Total	88	121	109	100	69	140	74	136	93	116
Sensitivity (95% CI)		95.0% (91.2% to 99.8%)		89.9% (84.2% to 95.6%),		91.3% (84.6 to 98.0%),		98.6% (95.9 to 100%)		57.0% (46.9 to 67.1%)	
Specificity (95% CI)		95.0% (92.4% to 99.4%)		98% (95.3% to 100%).		96.4% (93.3 to 99.5%)		99.3% (97.9 to 100%)		96.6% (93.3 to 99.9%)	
Overall agreement, (95% CI)		95.2 (92.9% to 98.5%).		93.8% (90.5% to 97.1%)		94.7% (91.7 to 97.7%)		99.0% (97.7% to 100%)		78.9% (73.4 to 84.4%)	

R: Resistance; S: Susceptible; CI: Confidence Interval. We investigated each drug by comparing the phenotype result of MGIT960 with the genotypic result of Sanger and sequencing and sensitivity specificity and accuracy are calculated as weighted means at 95% CIs.

The sensitivity of the INH resistance prediction was 89.9% (95%, [CI], 84.2% to 95.6%), and specificity was 98% (95%, CI, 95.3% to 100%). The overall agreement between the genotypic and phenotypic DST assays was 93.8% (95%, CI 90.5% to 97.1%) (Table 5). Discordant resistant results of two strains linked to *katG* S315T mutations (Table 4).

Regarding the prediction of EMB resistance, 74 strains were classified as having EMB resistance with a sensitivity of 98.6% (95% CI, 95.9 to 100%) and a specificity of 99.3% (95% CI, 97.9 to 100%). The overall agreement of the EMB resistant diagnosis was 99.0% (95% CI, 97.7% to 100%). One discordant resistant result was linked to the mutation *embB* D311G.

The sensitivity of the prediction of resistance to PZA by gDST was 91.3% (95% CI, 84.6 to 98.0%), whereas the specificity was 96.4% (9% CI, 93.3 to 99.5%). In addition, the overall correlation between pDST and gDST test results was found to be 94.7% (95% CI, 91.7 to 97.7%). However, the mutations in *pncA* A102V (3 strains), V130G (one strain), and V130M (one strain) were linked to the discordant resistance result. The sensitivity of STR resistance was 57.0% (95% CI, 46.9 to 67.1%) with a 96.6% (95% CI, 93.3 to 99.9%) specificity and an overall agreement of 78.9% (95% CI, 73.4 to 84.4%). Interestingly, no mutation in *rpsL* or *rrs* was found in 40 pDST resistant strains. In addition to this, four discordant streptomycin-resistant results were linked to the mutations in *rpsL* 88 and 906, (Table 4).

## Discussion

We employed Sanger sequencing of MTBC strains from MDR-TB suspects in Ethiopia to investigate the genomic mutations implicated in resistance against RIF, INH, PZA, EMB, and STR. Overall, Sanger sequencing showed high accuracy that ranged from 78.9% for detection of STR resistance to 99.0% for detection of EMB resistance when compared to the phenotypic standard method, the BACTEC MGIT 960 system. This implies that Sanger sequencing has the potential to predict first-line drug resistance among MDR-TB suspects and can be used as a complementary approach to pDST to detect low-level drug resistance in resource-limited countries.

Drug resistance TB is a very important public health threat globally. It is an alarming obstacle to TB care, treatment, and prevention, especially in resource-limited countries [28]. Moreover, it often leads to poor outcomes for TB patients [28]. In this study, the majority of the phenotypic resistance against RIF could be explained by mutations in the *rpoB* target region. The mutation in *rpoB* S531L was dominant and detected in 67% of the strains. This finding is similar to the findings of [28] in South Africa. However, in Sudan, a neighboring country to Ethiopia, resistance to RIF was mediated by the *rpoB* Ser450Leu, His445Tyr, His445Asn, and His445Asp mutations [29]. Moreover, we found mutations in the *rpoB* gene in six phenotypic RIF susceptible strains, including, four of the mutations detected within the RRDR at R529P (n = 1), L533P (n = 2), and S531S (n = 1) silent mutation) and two outside the RRDR at L538P/V. This could be explained by low-level RIF resistance [30]. The mutations detected within the RRDR at S531S and L533P have been associated with false resistance when using the probe-based gDST method (e.g., GeneXpert MTB/RIF), which may lead to the administration of unnecessary treatment (i.e., overtreatment) [30].

The most prevalent mutation conferring resistance against INH was *katG* S315T, and it was found in 97% of the MDR-TB strains. The *katG* S315T mutation has also been found to be dominant elsewhere in the world in countries like Sudan, South Africa, and Vietnam [29, 31, 32]. It is associated with a low-fitness cost but with clinically significant levels of INH resistance [32, 33]. Moreover, strains harboring the *katG* S315T mutation produce active catalase-peroxidase, tend to be in molecular clusters (i.e., transmissible from patient to patient), and are virulent in TB mouse models [33].

Furthermore, four of the five strains with INH resistance conferring mutations in the promoter region of the *fabG1*-inhA operon had an additional mutation at *katG* S315T. The co-occurrence of the *katG* S315T and the *fabG1*-inhA promoter mutations would lead to a further increase in the INH resistance level and render ethionamide or prothionamide treatment unsuccessful. Another possibility could be a compensatory effect of the *fabG1*-inhA promoter mutations in catalase deficient and INH resistant strains. The co-selection of the *fabG1*-inhA promoter mutations has also been observed in other studies [34–36].

Encouragingly, our Sanger sequencing approach, using the presence of mutations in the interrogated *embB* and *pncA* genes with regions, resulted in overall sensitivities (> 90%) and specificities (> 95%) for the prediction of EMB and PZA resistance. It is usually difficult to predict with genotypic tests of both drugs due to breakpoint artefacts in EMB resistant strains [37] and the diversity of *pncA* mutations in combination with challenging PZA test conditions [38–41].

Moreover, identical *pncA* mutations in MDR-TB strains from epidemiologically related patients might point towards ongoing transmission [42]. In this study, nearly 28% of the strains harbored the mutation *pncA* 64fs, while other patient strains showed very diverse and mostly unique *pncA* mutations. PZA is one of the essential drugs for the treatment of TB, including MDR TB [43]. However, currently, there is no reliable and rapid diagnostic method for the detection of PZA resistant TB, and the pDST method depends on acid PH and has a long turnaround time [43]. Thus, it is important to design or explore a reliable and rapid method. Interestingly, our findings showed that more than 97% of the genetic variants identified in the *pncA* gene were correlated with phenotypic resistance. Hence, Sanger sequencing could be a reliable and accurate method for the rapid diagnosis of PZA resistant TB [44, 45]. Regarding STR, the sensitivity of predicting resistance against this drug was most likely reduced due to the presence of a *gidB* mutation, which could not be interrogated in this study [46, 47].

## Conclusion

Overall, our study revealed that Sanger sequencing results can be used as a surrogate marker for pDST against all first-line drugs (INH, RIF, EMB, and PZA) in MDR-TB suspects with high accuracy. We showed that the sensitivity and specificity of this method are within the WHO recommendation for molecular assays. Moreover, Sanger sequencing is able to detect mutations that mediate only a low or moderate resistance increase. It detected many known canonical resistance-associated mutations implicated in resistance against RIF, INH, and EMB, as well as diverse mutations in the *pncA* gene associated with resistance against PZA. Further studies evaluating the performance of Sanger Sequencing to predict drug resistance profiles from direct patient specimens, e.g., sputum and other body fluids, are desirable. The ability to predict rare mutations (not covered by commercial molecular tests), especially *pncA* mutations and low-level resistance mutations, such as in *rpoB*, renders Sanger Sequencing a promising tool to complement routine pDST in MDR-TB suspects.

## Supporting information

S1 Table All socio-demographic and phenotypic and genotypic drug susceptibility test results data. (XLSX)

## References

[1] World Health Organization. Global tuberculosis report 2019. World Health Organization; 2019.

[2] Girum T, Muktar E, Lentiro K, Wondiye H, Shewangizaw M. Epidemiology of multidrug-resistant tuberculosis (MDR-TB) in Ethiopia: a systematic review and meta-analysis of the prevalence, determinants and treatment outcome. Tropical diseases, travel medicine and vaccines. 2018 Dec 1;4(1):5. doi: 10.1186/s40794-018-0065-5 29942536 PMC6000958

[3] Abate D, Taye B, Abseno M, Biadgilign S. Epidemiology of anti-tuberculosis drug resistance patterns and trends in tuberculosis referral hospital in Addis Ababa, Ethiopia. BMC research notes. 2012 Dec 1;5(1):462. doi: 10.1186/1756-0500-5-462 22929063 PMC3507648

[4] Abdella K, Abdissa K, Kebede W, Abebe G. Drug resistance patterns of Mycobacterium tuberculosis complex and associated factors among retreatment cases around Jimma, Southwest Ethiopia. BMC public health. 2015 Dec 1;15(1):599. doi: 10.1186/s12889-015-1955-3 26135909 PMC4489121

[5] Tessema B, Beer J, Emmrich F, Sack U, Rodloff AC. First-and second-line anti-tuberculosis drug resistance in Northwest Ethiopia. The International Journal of Tuberculosis and Lung Disease. 2012 Jun 1;16(6):805–11. doi: 10.5588/ijtld.11.0522 22390880

[6] Sinshaw W, Kebede A, Bitew A, Tesfaye E, Tadesse M, Mehamed Z, et al. Prevalence of tuberculosis, multidrug resistant tuberculosis and associated risk factors among smear negative presumptive pulmonary tuberculosis patients in Addis Ababa, Ethiopia. BMC Infectious Diseases. 2019 Dec 1;19(1):641. doi: 10.1186/s12879-019-4241-7 31324227 PMC6642575

[7] Mesfin EA, Beyene D, Tesfaye A, Admasu A, Addise D, Amare M, et al. Drug-resistance patterns of Mycobacterium tuberculosis strains and associated risk factors among multi drug-resistant tuberculosis suspected patients from Ethiopia. PloS one. 2018 Jun 4;13(6):e0197737 doi: 10.1371/journal.pone.0197737 29864118 PMC5986145

[8] Ilin AI, Kulmanov ME, Korotetskiy IS, Islamov RA, Akhmetova GK, Lankina MV, et al. Genomic insight into mechanisms of reversion of antibiotic resistance in multidrug resistant Mycobacterium tuberculosis induced by a nanomolecular iodine-containing complex FS-1. Frontiers in cellular and infection microbiology. 2017 May 8;7:151. doi: 10.3389/fcimb.2017.00151 28534009 PMC5420568

[9] Ong DC, Yam WC, Siu GK, Lee AS. Rapid detection of rifampicin-and isoniazid-resistant Mycobacterium tuberculosis by high-resolution melting analysis. Journal of clinical microbiology. 2010 Apr 1;48(4):1047–54. doi: 10.1128/JCM.02036-09 20164280 PMC2849564

[10] Piatek AS, Telenti A, Murray MR, El-Hajj H, Jacobs WR, Kramer FR, A et al. Genotypic analysis of Mycobacterium tuberculosis in two distinct populations using molecular beacons: implications for rapid susceptibility testing. Antimicrobial agents and chemotherapy. 2000 Jan 1;44(1):103–10. doi: 10.1128/AAC.44.1.103-110.2000 10602730 PMC89635

[11] Allix-Beguec C, Arandjelovic I, Bi L, Beckert P, Bonnet M, Bradley P, et al. Prediction of susceptibility to first-line tuberculosis drugs by DNA sequencing. New England Journal of Medicine. 2018 Oct 11;379(15):1403–15. doi: 10.1056/NEJMoa1800474 30280646 PMC6121966

[12] Feuerriegel S, Schleusener V, Beckert P, Kohl TA, Miotto P, Cirillo DM, et al. PhyResSE: a web tool delineating Mycobacterium tuberculosis antibiotic resistance and lineage from whole-genome sequencing data. Journal of clinical microbiology. 2015 Jun 1;53(6):1908–14. doi: 10.1128/JCM.00025-15 25854485 PMC4432036

[13] World Health Organization. The use of next-generation sequencing technologies for the detection of mutations associated with drug resistance in Mycobacterium tuberculosis complex: technical guide. World Health Organization; 2018.

[14] Schön T, Miotto P, Köser CU, Viveiros M, Böttger E, Cambau E. Mycobacterium tuberculosis drug-resistance testing: challenges, recent developments and perspectives. Clinical Microbiology and Infection. 2017 Mar 1;23(3):154–60. doi: 10.1016/j.cmi.2016.10.022 27810467

[15] Ajileye A, Alvarez N, Merker M, Walker TM, Akter S, Brown K, et al. Some synonymous and nonsynonymous gyrA mutations in Mycobacterium tuberculosis lead to systematic false-positive fluoroquinolone resistance results with the Hain GenoType MTBDRsl assays. Antimicrobial agents and chemotherapy. 2017 Apr 1;61(4). doi: 10.1128/AAC.02169-16 28137812 PMC5365657

[16] Federal Ministry of Health. Guideline for program and clinical management of drug-resistant tuberculosis. Addis Ababa; 2009

[17] Narvaiz I, Kim KS, Frieden T, Laszlo A, Luelmo F, Norval PY, et al. Laboratory services in tuberculosis control. WHO, Geneva, Switzerland. 1998.

[18] Siddiqi SH, Rüsch-Gerdes S. MGIT procedure manual. Foundation for Innovative New Diagnostics, Geneva, Switzerland. 2006 Jul:41–51.

[19] Kent PT. Public health mycobacteriology: a guide for the level III laboratory. US Department of Health and Human Services, Public Health Service, Centers for Disease Control; 1985.

[20] European Centre for Disease Prevention and Control. Handbook on TB laboratory diagnostic methods for the European Union. Stockholm, ECDC; 2016.

[21] Somerville W, Thibert L, Schwartzman K, Behr MA. Extraction of Mycobacterium tuberculosis DNA: a question of containment. Journal of clinical microbiology. 2005 Jun 1;43(6):2996–7. doi: 10.1128/JCM.43.6.2996-2997.2005 15956443 PMC1151963

[22] Homolka S, Meyer CG, Hillemann D, Owusu-Dabo E, Adjei O, Horstmann RD, et al. Unequal distribution of resistance-conferring mutations among Mycobacterium tuberculosis and Mycobacterium africanum strains from Ghana. International Journal of Medical Microbiology. 2010 Nov 1;300(7):489–95. doi: 10.1016/j.ijmm.2010.04.019 20538518

[23] Zhao JR, Bai YJ, Wang Y, Zhang QH, Luo M, Yan XJ. Development of a pyrosequencing approach for rapid screening of rifampin, isoniazid and ethambutol-resistant Mycobacterium tuberculosis. The International Journal of Tuberculosis and Lung Disease. 2005 Mar 1;9(3):328–32. 15786899

[24] Telenti A, Honore N, Bernasconi CA, March J, Ortega A, Heym B, et al. Genotypic assessment of isoniazid and rifampin resistance in Mycobacterium tuberculosis: a blind study at reference laboratory level. Journal of clinical microbiology. 1997 Mar 1;35(3):719–23. doi: 10.1128/jcm.35.3.719-723.1997 9041419 PMC229657

[25] Khan SN, Niemann S, Gulfraz M, Qayyum M, Siddiqi S, Mirza ZS, et al. Molecular characterization of multidrug-resistant strains of Mycobacterium tuberculosis from patients in Punjab, Pakistan. Pakistan Journal of Zoology. 2013 Feb 1;45(1):93–100.

[26] Sreevatsan S, Pan X, Stockbauer KE, Williams DL, Kreiswirth BN, Musser JM. Characterization of *rpsL* and *rrs* mutations in streptomycin-resistant Mycobacterium tuberculosis strains from diverse geographic localities. Antimicrobial agents and chemotherapy. 1996 Apr 1;40(4):1024–6. doi: 10.1128/AAC.40.4.1024 8849220 PMC163252

[27] Koivula T, Ekman M, Leitner T, Löfdahl S, Ghebremicahel S, Mostowy S, et al. Genetic characterization of the Guinea-Bissau family of Mycobacterium tuberculosis complex strains. Microbes and infection. 2004 Mar 1;6(3):272–8. doi: 10.1016/j.micinf.2003.12.006 15026014

[28] Rukasha I, Said HM, Omar SV, Koornhof H, Dreyer AW, Musekiwa A, et al. Correlation of rpoB mutations with minimal inhibitory concentration of rifampin and rifabutin in Mycobacterium tuberculosis in an HIV/AIDS endemic setting, South Africa. Frontiers in microbiology. 2016 Dec 5;7:1947. doi: 10.3389/fmicb.2016.01947 27994580 PMC5136537

[29] Shuaib YA, Khalil EA, Wieler LH, Schaible UE, Bakheit MA, Mohamed-Noor SE, et al. Mycobacterium tuberculosis complex lineage 3 as causative agent of pulmonary tuberculosis, eastern Sudan. Emerging infectious diseases. 2020 Mar;26(3):427. doi: 10.3201/eid2603.191145 32091355 PMC7045825

[30] Shea J, Halse TA, Kohlerschmidt D, Lapierre P, Modestil HA, Kearns CH, et al. Low-level rifampin resistance and *rpoB* mutations in Mycobacterium tuberculosis: an analysis of whole-genome sequencing and drug susceptibility test data in New York. Journal of Clinical Microbiology. 2020 Sep 30;59(4):e01885–20. 10.1128/JCM.01885-20 PMC8579749 32999007

[31] Cohen KA, Abeel T, Manson McGuire A, Desjardins CA, Munsamy V, Shea TP, et al. Evolution of extensively drug-resistant tuberculosis over four decades: whole genome sequencing and dating analysis of Mycobacterium tuberculosis isolates from KwaZulu-Natal. PLoS medicine. 2015 Sep 29;12(9):e1001880. doi: 10.1371/journal.pmed.1001880 26418737 PMC4587932

[32] Le Hang NT, Hijikata M, Maeda S, Thuong PH, Ohashi J, Van Huan H, et al. Whole genome sequencing, analyses of drug resistance-conferring mutations, and correlation with transmission of Mycobacterium tuberculosis carrying katG-S315T in Hanoi, Vietnam. Scientific reports. 2019 Oct 25;9(1):1–4. 31653940 10.1038/s41598-019-51812-7 PMC6814805

[33] Pym AS, Saint-Joanis B, Cole ST. Effect of *katG* mutations on the virulence of Mycobacterium tuberculosis and the implication for transmission in humans. Infection and immunity. 2002 Sep;70(9):4955–60. doi: 10.1128/IAI.70.9.4955-4960.2002 12183541 PMC128294

[34] Casali N, Nikolayevskyy V, Balabanova Y, Harris SR, Ignatyeva O, Kontsevaya I, et al. Evolution and transmission of drug-resistant tuberculosis in a Russian population. Nature genetics. 2014 Mar 46(3):279–86. doi: 10.1038/ng.2878 24464101 PMC3939361

[35] Eldholm V, Monteserin J, Rieux A, Lopez B, Sobkowiak B, Ritacco, et al. Four decades of transmission of a multidrug-resistant Mycobacterium tuberculosis outbreak strain. Nature communications. 2015 May 11;6(1):1–9. doi: 10.1038/ncomms8119 25960343 PMC4432642

[36] Klopper M, Heupink TH, Hill-Cawthorne G, Streicher EM, Dippenaar A, De Vos M, et al,. A landscape of genomic alterations at the root of a near-untreatable tuberculosis epidemic. BMC medicine. 2020 Dec 18(1):1–4. 32014024 10.1186/s12916-019-1487-2 PMC6998097

[37] Ängeby K, Juréen P, Kahlmeter G, Hoffner SE, Schön T. Challenging a dogma: antimicrobial susceptibility testing breakpoints for Mycobacterium tuberculosis. Bulletin of the World Health Organization. 2012;90:693–8. doi: 10.2471/BLT.11.096644 22984314 PMC3442398

[38] Zhang Y, Permar S, Sun Z. Conditions that may affect the results of susceptibility testing of Mycobacterium tuberculosis to pyrazinamide. Journal of medical microbiology. 2002 Jan 1;51(1):42–9. doi: 10.1099/0022-1317-51-1-42 11800471

[39] Chang KC, Yew WW, Zhang Y. Pyrazinamide susceptibility testing in Mycobacterium tuberculosis: a systematic review with meta-analyses. Antimicrobial agents and chemotherapy. 2011 Oct;55(10):4499–505. doi: 10.1128/AAC.00630-11 21768515 PMC3186960

[40] Hirano K, Takahashi M, Kazumi Y, Fukasawa Y, Abe C. Mutation in *pncA* is a major mechanism of pyrazinamide resistance in Mycobacterium tuberculosis. Tubercle and Lung Disease. 1998 Jan 1;78(2):117–22. 10.1016/s0962-8479(98)80004-x 9692180

[41] Cui Z, Wang J, Lu J, Huang X, Zheng R, Hu Z. Evaluation of methods for testing the susceptibility of clinical Mycobacterium tuberculosis isolates to pyrazinamide. Journal of clinical microbiology. 2013 May 1;51(5):1374–80 doi: 10.1128/JCM.03197-12 23390285 PMC3647927

[42] Walker TM, Merker M, Knoblauch AM, Helbling P, Schoch OD, Van Der Werf MJ, et al. A cluster of multidrug-resistant Mycobacterium tuberculosis among patients arriving in Europe from the Horn of Africa: a molecular epidemiological study. The Lancet Infectious Diseases. 2018 Apr 1;18(4):431–40. doi: 10.1016/S1473-3099(18)30004-5 29326013 PMC5864516

[43] World Health Organization. World Health Organization Global Tuberculosis Report 2020. World Health Organization. 2020;232.

[44] Miotto P, Cabibbe AM, Feuerriegel S, Casali N, Drobniewski F, Rodionova Y, et al. Mycobacterium tuberculosis pyrazinamide resistance determinants: a multicenter study. MBio. 2014 Oct 31;5(5). doi: 10.1128/mBio.01819-14 25336456 PMC4212837

[45] Havlicek J, Dachsel B, Slickers P, Andres S, Beckert P, Feuerriegel S, et al. Rapid microarray-based assay for detection of pyrazinamide resistant Mycobacterium tuberculosis. Diagnostic microbiology and infectious disease. 2019 Jun 1;94(2):147–54 doi: 10.1016/j.diagmicrobio.2018.12.011 30733004 PMC6531379

[46] Wong SY, Lee JS, Kwak HK, Via LE, Boshoff HI, Barry CE III. Mutations in gidB confer low-level streptomycin resistance in Mycobacterium tuberculosis. Antimicrobial agents and chemotherapy. 2011 Jun;55(6):2515–22. doi: 10.1128/AAC.01814-10 21444711 PMC3101441

[47] Verma JS, Gupta Y, Nair D, Manzoor N, Rautela RS, Rai A, et al. Evaluation of gidB alterations responsible for streptomycin resistance in Mycobacterium tuberculosis. Journal of Antimicrobial Chemotherapy. 2014 Nov 1;69(11):2935–41. doi: 10.1093/jac/dku273 25074855

